# EP-Pred: A Machine Learning Tool for Bioprospecting Promiscuous Ester Hydrolases

**DOI:** 10.3390/biom12101529

**Published:** 2022-10-21

**Authors:** Ruite Xiang, Laura Fernandez-Lopez, Ana Robles-Martín, Manuel Ferrer, Victor Guallar

**Affiliations:** 1 Department of Life Sciences, Barcelona Supercomputing Center (BSC), 08034 Barcelona, Spain; 2 Department of Applied Biocatalysis, ICP, CSIC, 28049 Madrid, Spain; 3 Catalan Institution for Research and Advanced Studies (ICREA), 08010 Barcelona, Spain

**Keywords:** biocatalysts, bioprospecting, esterases/lipases, hydrolases, machine learning, supervised learning

## Abstract

When bioprospecting for novel industrial enzymes, substrate promiscuity is a desirable property that increases the reusability of the enzyme. Among industrial enzymes, ester hydrolases have great relevance for which the demand has not ceased to increase. However, the search for new substrate promiscuous ester hydrolases is not trivial since the mechanism behind this property is greatly influenced by the active site’s structural and physicochemical characteristics. These characteristics must be computed from the 3D structure, which is rarely available and expensive to measure, hence the need for a method that can predict promiscuity from sequence alone. Here we report such a method called EP-pred, an ensemble binary classifier, that combines three machine learning algorithms: SVM, KNN, and a Linear model. EP-pred has been evaluated against the Lipase Engineering Database together with a hidden Markov approach leading to a final set of ten sequences predicted to encode promiscuous esterases. Experimental results confirmed the validity of our method since all ten proteins were found to exhibit a broad substrate ambiguity.

## 1. Introduction

Enzymes are of great interest for a vast majority of industries, partially due to the increasing concerns over environmental issues. Among the many classes of enzymes, hydrolases stand out for their industrial relevance because of their high stereoselectivity, commercial availability and stability in organic solvents [1]. Indeed, the demand for newer and better hydrolases that can work in industrial settings has only increased exponentially over the years. Specifically, ester hydrolases (EC 3.1), which hydrolyze ester bonds, have received considerable attention, and are extensively used in various areas such as food, detergents, agriculture, pharmaceuticals, and so on [2]. 

Searching for new esterase candidates is not trivial, in fact, there are strict requirements regarding stability, activity and substrate promiscuity which are difficult to find conjointly in natural enzymes [3]. Actually, one of the most common issues that an industrial enzyme will face is having low substrate promiscuity [4]; the ability to catalyze a specific reaction for a variety of different substrates. It is a desirable characteristic since one single enzyme could be used for multiple applications, thus reducing the cost and time of development and production of multiple biocatalysts [5]. 

While some substrate promiscuous enzymes might suffer from limited stereoselectivity and lower catalytic rates [6,7], they are typically enzymes prone to accept enzyme engineering, which could restore these properties. In previous studies, we have investigated the determinants of substrate ambiguity for esterases at a molecular level, establishing rules for its prediction [4], and introducing a significant increase in the number of substrates hydrolyzed through engineering [8]. However, it must be noted that the necessary molecular metrics must be computed from the 3D structures which are rarely available, and that they involve significant computational time. Even with the recent advancements in the accuracy of deep learning structural predictions, such as AlphaFold 2.0 [9], it is still unfeasible to analyze substrate specificity from the ever growing number of annotated sequences. For instance, as of 9 March 2021, the Lipase Engineering Database (LED) [10], which holds data on esterases/lipases and a few other homologous sequences, contains about 280,638 entries. In addition, AlphaFold 2.0 models tend to generate APO structures, with a significant modification (mostly volume reduction) in the active site that precludes, for example, efficient ligand rigid docking [11]. These changes would largely affect promiscuity predictions using the molecular descriptors. 

Therefore, methods to directly identify substrate promiscuity from sequence alone would greatly increase the efficiency of bioprospecting for new esterase candidates, a task ideal for machine learning algorithms (see for example, other recently developed methods for activity predictions [12]). Several studies have already predicted enzyme substrate promiscuity using molecular descriptors [13] or machine learning [14,15,16] approaches, although for other enzyme families. In addition, there are several differences in our approach. First, other studies mainly used training samples listed in databases such as the Kyoto Encyclopedia of Genes and Genomes (KEGG), which includes reaction data for most of the studied enzyme families. In these databases the number of tested compounds is usually not large, hindering the correct identification of promiscuous enzymes. Additionally, those studies that revolved around single enzyme families are usually constrained by the number of samples from which to derive the molecular descriptors or the classification models which might decrease their applicability. Second, the goal of the developed methods seems to differ. Previous projects evaluate whether a specific compound will be catalyzed by a set of characterized enzymes or the reverse to find novel substrates or enzymes. Our approach, in addition, tries to classify how promiscuous an enzyme might be in a bioprospecting setting. 

Here we report the development and application of an ensemble binary classifier trained on a dataset of 145 diverse esterases and 96 substrates [4], using a combination of physicochemical and evolutionary feature vectors extracted from the primary sequence. Our classifier, named EP-pred, combines three types of classification algorithms: SVM (support vector machines), KNN (k-nearest neighbors) and RidgeClassifier, one of the linear models implemented in Scikit-Learn. EP-pred was then evaluated against LED and from those predicted to be positives, a final set of ten sequences were isolated and tested experimentally. All selected enzymes were confirmed to be substrate promiscuous, highlighting the potential of our machine learning bioprospecting method.

## 2. Materials and Methods

### 2.1. Esterase Dataset

The dataset employed to train the models is the same used in our previous molecular modeling studies and it is formed by 145 diverse microbial ester hydrolases with pairwise sequence identities ranging from 0.2 to 99.7% and an average pairwise identity of 13.7% [4]. The heterogeneity of the sequences can be attributed to the diversity of the source from which they were isolated, including both terrestrial bacteria from 28 geographically distinct sites and marine bacteria. The phylogenetic analysis performed in the previous study further supports the diversity of the source bacteria since they were found to be distributed across the phylogenetic tree. 

The substrate profiles of the enzymes were assessed on a set of 96 diverse esters with the most promiscuous one capable of hydrolyzing 72 esters out of 96 tested and the least promiscuous one only capable of catalyzing 1 out of 96 substrates. The distinction between promiscuous and not promiscuous, or positive and negative classes, was also established according to the threshold of the previous study: 20 substrates.

### 2.2. Lipase Engineering and Uniref 50 Databases

The lipase engineering database (https://led.biocatnet.de/, accessed on 9 March 2021), used to evaluate the final model, gathers information on the sequence, structure and function of esterases/lipases and other related proteins sharing the same a/b hydrolase fold. The whole sequence database was downloaded in Fasta format containing 280,638 entries.

The evolutionary-related features were based on PSSM (Position Specific Scoring Matrix) profiles which were generated with Psi-Blast by querying the input sequence against the Uniref 50. Uniref or The UniProt Reference Clusters contains records of the Uniprot Knowledgebase and the Uniparc sequence archive at several resolutions, 100%, 90% and 50%, each one generated from the clustering of the previous one. Thus, Uniref50 is generated from clustering the UniRef90 seed sequences at the identity threshold of 50% [17].

### 2.3. Feature Extraction

Two web servers Possum [18] and iFeature [19] were used to extract evolutionary information and physicochemical properties, respectively, from all protein sequences. iFeature can generate 53 different types of descriptors, from which 32 were extracted using the default parameters resulting in a total of 2274 feature vector dimensions. The rest of the feature types were discarded because they can only be applied to sequences of the same length. These features might be simplistic descriptors like the amino acid composition (AAC), which counts the frequency of each amino acid in the sequence. However, there are also more elaborate forms of descriptors that account for the distribution, transition, or correlation of different properties, like hydrophobicity, along the sequence.

Possum generates features based on the PSSM (Position Specific Scoring Matrix) profiles that contain evolutionary information of the sequences since it specifies the scores for observing particular amino acids at specific positions of the sequence. Although very informative, the downside of these profiles is that they depend on the length of the sequences which hampers their direct use as features for machine learning applications. By applying different matrix transformations to make them length-independent, Possum was able to generate 18 different descriptors that were extracted resulting in a vector of 18,730 dimensions. Some transformations are inspired by sequence-based features, such as ACC which reduces the PSSM profile from a matrix of L × 20 dimensions, L being the length of a sequence, to a vector of 20 dimensions by averaging the scores of the rows in the PSSM. While other transformations are more complex constructs that first scale, filter, or group the values in the PSSM and then apply various operations to fix the dimensions of the matrix.

The concatenation of both feature vectors yields a feature set of 21,000 dimensions for the esterase dataset. After generating the features, some cleaning was needed because many columns had zeros or identical values in most of the rows which carried little information. As a result, 2274 iFeature and 18,730 Possum features were reduced to 1203 and 14,606 dimensions, respectively.

### 2.4. Feature Selection

Even with the cleaning, the number of original features remained exceedingly high, therefore feature selection was needed to eliminate noise and avoid overfitting. As recommended for this step [20], data was split into two sets, a test set and a training set and the selection was performed on the training set only. In addition, the number of dimensions was reduced to less than ½ of the number of samples as it was shown to reduce overfitting.

It must be noted that the dimensionality reduction of iFeature and Possum descriptors was carried out independently and concatenated later to generate the features. However, the proportion of the two descriptors was not even because evolutionary-related features seem to bear more information [18,21,22], so they were given a larger weight during the construction of the feature set compared to the iFeature descriptors. Furthermore, following this idea, five other sets of features with varying dimensions were also constructed and tested. The whole process was repeated ten times, one for each of the selection algorithms resulting in a total of 60 feature sets.

The selection methods could be divided into three categories: filter methods that assessed the degree of dependence between the features and the labels, wrapper and embedded methods that applied machine learning algorithms to rank those features based on their relevance for the performance [23].

A total of 5 libraries were used to implement the methods from the different categories: (I) ITMO_FS [24] which provided filter methods such as Chi-square and Information gain. (II) Boruta [25] a library containing a single embedded method. (III) Scikit-feature [26] that implemented the filter methods MRMR (minimum redundancy maximum relevancy) and CIFE (conditional infomax feature extraction). (IV) Scikit-learn that provided the filter methods mutual information and fisher score; the wrapper method RFE (recursive feature elimination) combined with a linear model or SVM and the embedded method random forest. Finally, (V) XGBoost [27], like boruta, is a library that contains only an embedded method.

### 2.5. Model Training

SVM, KNN and RidgeClassifier, which are all implemented in Scikit-Learn, were selected for classification. To correctly evaluate the model’s performance, we employed a similar strategy to nested cross-validation [20]. The data was split into a 20% test set and 80% training set 5 times, each time generating different sets. Then, for each split, the training set was used for model development to find the optimal sets of hyperparameters using 5-fold cross-validation while the test set was used for the evaluation. It generates five measurements from models with different sets of hyperparameters that can be used to compute the statistics on the model’s performance.

### 2.6. Performance Metrics

Using the TP (true positive), TN (true negative), FN (false negative) and FP (false positive) values, the precision (Pr), recall (Re), F1 [28] and Matthew’s correlation coefficient (MCC) were calculated [29] to evaluate the performance of the models.

(1) $$\mathrm{Pr}=\frac{\mathrm{TP}}{\mathrm{FP}+\mathrm{TP}}$$

(2) Re=TPFN+TP 

(3) F1=2∗ Pr∗RePr+ Re

(4) $$\mathrm{MCC}=\frac{\mathrm{TP}*\;\mathrm{TN}-\mathrm{FP}*\mathrm{FN}}{\sqrt{\left(\mathrm{TP}+\mathrm{FP}\right)*\left(\mathrm{TP}+\mathrm{FN}\right)*\left(\mathrm{TN}+\mathrm{FP}\right)*\left(\mathrm{TN}+\mathrm{FN}\right)}}$$

### 2.7. Applicability Domain

There are several aspects that might affect the reliability of the model’s predictions apart from the performance metrics. Indeed, there should be limitations in the applicability of the models to be used only on those samples that are similar to the training samples, because otherwise, it would be predicting sequences that it has not seen and fitted before. In other words, we should define the applicability domain (AD) of the models and filter the predictions accordingly.

There are several approaches to define the similarity or the AD, all within the feature or descriptor space, but we decided to follow one inspired by KNN [30]. In this approach, a distance threshold was computed for each training sample and compared to the Euclidean distance between a new sample and each training sample. If any of the distances between the new and the training samples was less or equal to the threshold associated with that training sample, the prediction was deemed reliable and kept.

### 2.8. Hidden Markov Model (HMM) Profiles

LED contains sequences other than esterases/lipases so it would be wise to filter them and keep only those most likely to be esterases before the predictions. For this purpose, we employed the HMM profiles, probabilistic models that capture the evolutionarily conserved patterns revealed by multiple sequence alignments (MSA). They allow more sensitive homology searches than blast while retaining the speed.

We followed the protocol described by Pérez-García et al [31]. The program used to build such profiles was HMMER (http://hmmer.org/, accessed on 10 March 2021), using a MSA of the esterases with 35 or more substrates. The MSA was generated by T-Coffee with the default parameters. The program can then use the HMM profile to search for homologs in sequence databases and filter them based on E-values; here we used an E-value cutoff of 10^−10^ to add precision. Notice that the resulting HMM model is aimed at filtering esterases rather than promiscuity. In fact, when applied to the training dataset, it cannot distinguish well between promiscuous and non-promiscuous enzymes, with a precision score of 0.6 at E-value of 0.001.

### 2.9. Homology Modelling (HM) and Active Site Analysis

The top selected predictions were modeled using ModWeb [32], a web server for protein structure modeling, which automatically generates a homology model of the target sequence. Note that here we only aimed at a fast structural method to generate approximate active site structures so we could discard clearly wrong ones. Notice that for those sequences tested experimentally, we also constructed the Alphafold2 models (not yet available at the conception of the project). AlphaFold2 results clearly agreed with the ones predicted by ModWeb, with low values of RMSD when compared (see, Table S6). We also checked the structural quality of the models from the top 10 promiscuous esterases with ProSA-web [33]. It compares the structure models with experimentally determined proteins from Protein Data Bank and estimates a Z-score for each model, the lower the better.

The active site of the homology models was analyzed to filter out esterases with the catalytic triads not arranged in an active conformation to make sure they were indeed esterases. Next, the properties of their active site were calculated using SiteMap, Schrodinger [34,35] which includes hydrophobicity, enclosure and exposure that gave an idea of how solvent-exposed the cavity of the enzymes was. These metrics are relevant because we found out [8] that the active site of the promiscuous esterases share some common physicochemical features such as high hydrophobicity, larger volumes and are more enclosed compared to their non-promiscuous counterparts. Accordingly, we used this information to rank and isolate the 10 sequences tested in this paper when we needed to reduce the number of candidates.

### 2.10. Enzyme Source, Production, and Purification

The sequences encoding AJP48854.1, ART39858.1, PHR82761.1, WP_014900537.1, WP_026140314.1, WP_042877612.1, WP_059541090.1, WP_069226497.1, WP_089515094.1 and WP_101198885.1 were used as templates for gene synthesis (GenScript Biotech, EG Rijswijk, The Netherlands), and genes were codon-optimized to maximize expression in *Escherichia coli*. Genes were flanked by BamHI and HindIII (stop codon) restriction sites and inserted in a pET-45b(+) expression vector with an ampicillin selection marker (GenScript Biotech, Rijswijk, The Netherlands), which was further introduced into *E. coli* BL21(DE3).

The soluble N-terminal histidine (His) tagged proteins were produced and purified (98% purity, as determined by SDS–PAGE analysis using a Mini PROTEAN electrophoresis system, Bio-Rad, Madrid, Spain) at 4 °C after binding to a Ni-NTA His-Bind resin (Merck Life Science S.L.U., Madrid, Spain), as previously described [4,7], and stored at −86 °C until use at a concentration of 10 mg mL^−1^ in 40 mM 4-(2-hydroxyethyl)-1-piperazineethanesulfonic acid (HEPES) buffer (pH 7.0).

### 2.11. Activity Tests

The hydrolysis of esters was assayed using a pH indicator assay in 384-well plates (ref. 781162, Greiner Bio-One GmbH, Kremsmünster, Austria) at 40 °C and pH 8.0 in a Synergy HT Multi-Mode Microplate Reader in continuous mode at 550 nm over 24 h (extinction coefficient (ε) of phenol red, 8450 M^−1^ cm^−1^), as reported [36,37]. The conditions for determining the specific activity (units mg^−1^) were as follows: [proteins]: 270 μg mL^−1^; [ester]: 20 mM; reaction volume: 44 μL; T: 30 °C; and pH: 8.0 (5 mM 4-(2-hydroxyethyl)-1-piperazinepropanesulfonic acid (EPPS) buffer). In all cases, all values in triplicate were corrected for nonenzymatic transformation, with the absence of activity defined as having at least a twofold background signal. In all cases, the activity was calculated by determining the absorbance per minute from the slopes generated [38].

The activity toward the model esters *p*-nitrophenyl (*p*-NP) acetate (ref. N-8130; Merck Life Science S.L.U., Madrid, Spain), propionate (Santa Cruz Biotechnology, Inc., Heidelberg, Germany, ref. sc-256813) and butyrate (ref. N-9876; Merck Life Science S.L.U., Madrid, Spain) was assessed in 5 mM EPPS buffer at pH 8.0 and 30 °C by monitoring the production of 4-nitrophenol at 348 nm (pH-independent isosbestic point, ε = 4147 M^−1^ cm^−1^) over 5 min and determining the absorbance per minute from the generated slopes [36]. The reactions were performed in 96-well plates (ref. 655801, Greiner Bio-One GmbH, Kremsmünster, Austria). For specific activity determinations, the following conditions were used: [proteins]: 7 μg mL^−1^; [ester]: 1 mM; reaction volume: 200 μL; T: 30 °C; and pH: 8.0 (5 mM EPPS buffer). For *K*_m_ and *k*_cat_ determinations (using *p*-NP propionate), the following conditions were used: [proteins]: 0.06–25 μg mL^−1^; [ester]: 0–0.04 mM; reaction volume: 100 μL; T: 30 °C; and pH: 8.0 (5 mM EPPS buffer). The values correspond to the fit obtained from the regression of the data (each obtained in triplicates) using SigmaPlot 14.0 software. *k*_cat_ was calculated by using the following equation: *k*_cat_ = V_max_/[E], where [E] = total enzyme (for raw data see Supplementary Material).

Meta-cleavage product (MCP) hydrolase activity was assayed using 2-hydroxy-6-oxo-6-phenylhexa-2,4-dienoate (HOPHD) and 2-hydroxy-6-oxohepta-2,4-dienoate (HOHD), freshly produced as described [38]. The reactions were performed at 30 °C in 96-well plates (ref. 655801, Greiner Bio-One GmbH, Kremsmünster, Austria), and they contained 7.0 μg mL^−1^ proteins and 0.2 mM HOPHD or HODH in a total volume of 200 μL 50 mM K/Na-phosphate (pH 7.5) buffer (this buffer was shown to be optimal for measuring MCP hydrolytic activity [38]. Hydrolysis was monitored at 388 nm (for HOPHD) or 434 nm (for HOHD) over 5 min and the absorbance per minute was determined from the generated slopes [38].

## 3. Results and Discussion

### 3.1. Model Buildup

The accuracy of machine learning classifiers depends greatly on the features and the hyperparameters used. To construct the features, we derived physicochemical and evolutionary information from the sequences via two webservers iFeatures [19] and Possum [18] (see Tables S1 and S2, respectively), reduced their dimension through feature selection and built a total of 60 sets of features to be tested.

**Figure 1:**
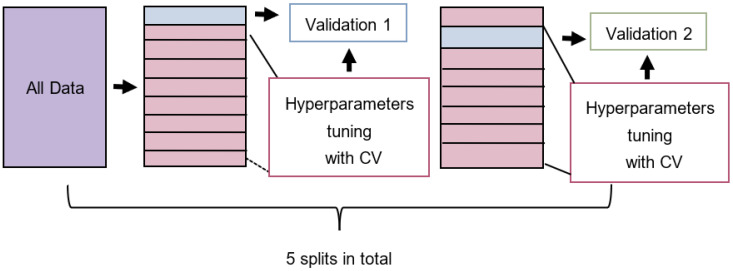
Graphical representation of the model training process. Data was split five times into different test and training sets. The training set was then used for tuning the hyperparameters using 5-fold cross-validation (CV) while the test set was used to evaluate the trained models.

**Figure 2:**
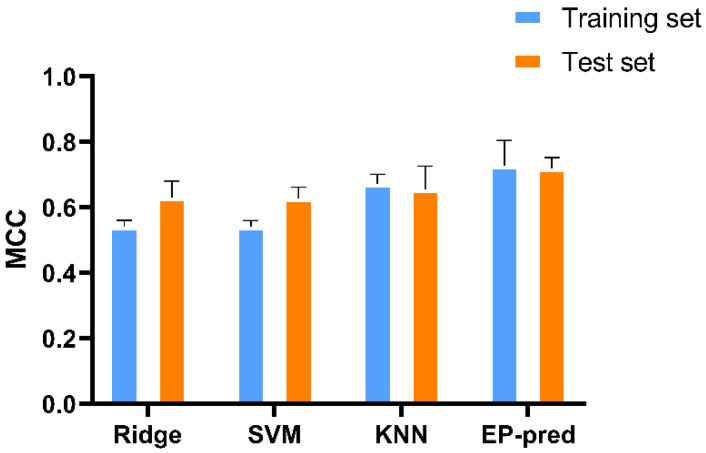
Matthew’s correlation coefficient (MCC) scores of the different classifiers. Ridge is the RidgeClassifier which is one of the linear models implemented in Scikit-Learn; SVM is the support vector machine; KNN is the K-nearest neighbors and EP-pred is the ensemble classifier that combined all 3 of the previous classifiers.

The classifiers were then trained on one of the feature sets during which the hyperparameters were tuned using 5-fold cross-validation (CV). Lastly, the model with the optimal hyperparameters was evaluated on the test set. The process was repeated five times, one for each of the data splits, from which the statistics on the model performance were computed and compared against other models using a distinct feature set (Figure 1). Among the 60 features, two sets generated the best models, called hereafter ch_20 and random_30, since they were produced by Chi-squared and Random Forest feature selection methods, respectively. Both SVM and RidgeClassifier performed the best when trained on ch_20 and both showed a mean MCC score of 0.54 for the training set and a mean MCC score of around 0.62 for the test set. In contrast, KNN performed the best when trained on random_30 and showed a mean MCC score of 0.67 for the training set and a mean MCC score of 0.65 for the test set (Figure 2). KNN slightly outperformed the others in the training set score which might imply that the algorithm might be more suited at fitting to this type of data.

PSSM-based features, which encode the evolutionary information of the sequences, seem to be more relevant for the prediction accuracy. In both selected sets, PSSM features proportion was larger than physicochemical ones. For instance, 19 out of 25 descriptors in random_30 were extracted from PSSM. This is in line with our previous findings [4] where phylogeny was a predictive marker of the substrate promiscuity in ester hydrolases.

The MCC scores for the three classifiers, which indicate the correlation between the predicted and the true labels, are good, but different models can be grouped to improve the performance since they have different biases that might complement each other. There are many possible combinations because each machine learning algorithm was trained on five different data sets resulting in five distinct classifiers. However, to generate the combinations, only two to three models from each algorithm were chosen, those with better MCC scores. EP-pred, which aggregates all the models, two SVM, three RidgeClassier and two KNN models, displayed a mean MCC score of 0.73 for the training set and a score of 0.72 for the test set (Figure 2). The observed increase in the models’ scores can be attributed to the fact that only samples for which the predictions between different classifiers agreed were kept for scoring, thus making the predictions more robust.

### 3.2. The Workflow for In Silico Bioprospecting

LED gathers sequences from various families apart from esterases/lipases, which is why we applied an HMM profile, built from the esterase dataset, as a filtering step and ended up with approximately 70,000 sequences. Then, the final model EP-pred was evaluated against them and predicted around 500 positive (promiscuous) sequences which were still too much for the experimental validation. Thus, several filters were applied to decrease the number of hits to a final set of ten.

**Figure 3:**
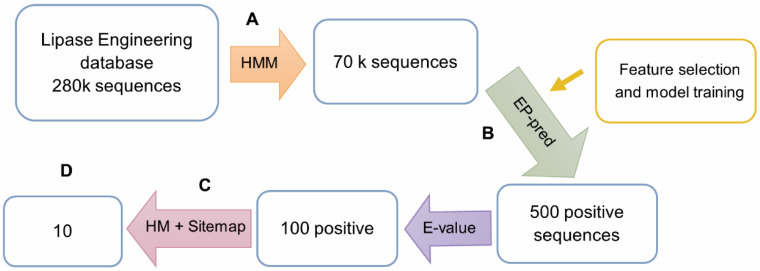
A description of the bioprospecting workflow. A, Since there was a mix of different families in LED, first we applied an HMM profile created from the esterase dataset to clean the database and keep only esterases. B, EP-pred evaluated the remaining sequences and predicted around 500 positive hits. C, The top 100 sequences according to E-values returned by HMM in step A were isolated and analyzed according to molecular descriptors from homology modeling (HM) and Sitemap calculations. D, A final set of 10 sequences with the highest hydrophobicity and enclosure/exposure scores were gathered and sent to be validated experimentally.

The top 100 sequences according to E-values returned by HMM were selected to be modeled and their active site cavity analyzed in search of the catalytic triad and geometric descriptors. Only 73 sequences passed this second filter and were forwarded to the subsequent analysis by SiteMap, a widely used binding site analysis tool, which then generated various binding cavity descriptors. As seen in our previous engineering studies, two metrics: hydrophobicity, and the ratio of enclosure/exposure, were useful in ranking promiscuity, see Table S3; thus, we used these to rank the final set of ten proteins for experimental validation picking those that intersected at the top in both metrics (Figure 3).

### 3.3. The Experimental Validation

All ten recombinant presumptive substrate promiscuous hydrolases (AJP48854.1, ART39858.1, PHR82761.1, WP_014900537.1, WP_026140314.1, WP_042877612.1, WP_059541090.1, WP_069226497.1, WP_089515094.1 and WP_101198885.1) were successfully expressed in soluble form and purified by nickel affinity chromatography. Then, three model p-nitrophenyl (*p*-NP) ester substrates with different chain lengths: *p*-NP acetate (C_2_), *p*-NP propionate (C_3_), and *p*-NP butyrate (C_4_) were first used to determine the substrate specificity of the enzymes. Their hydrolytic activity was assessed and recorded under standard assay conditions described in Section 2.11. We found specific activities ranging from 5.85 U mg^−1^ to 2.19 U mg^−1^ for *p*-NP propionate, which was the best substrate in all cases (Table 1).

**Figure 4:**
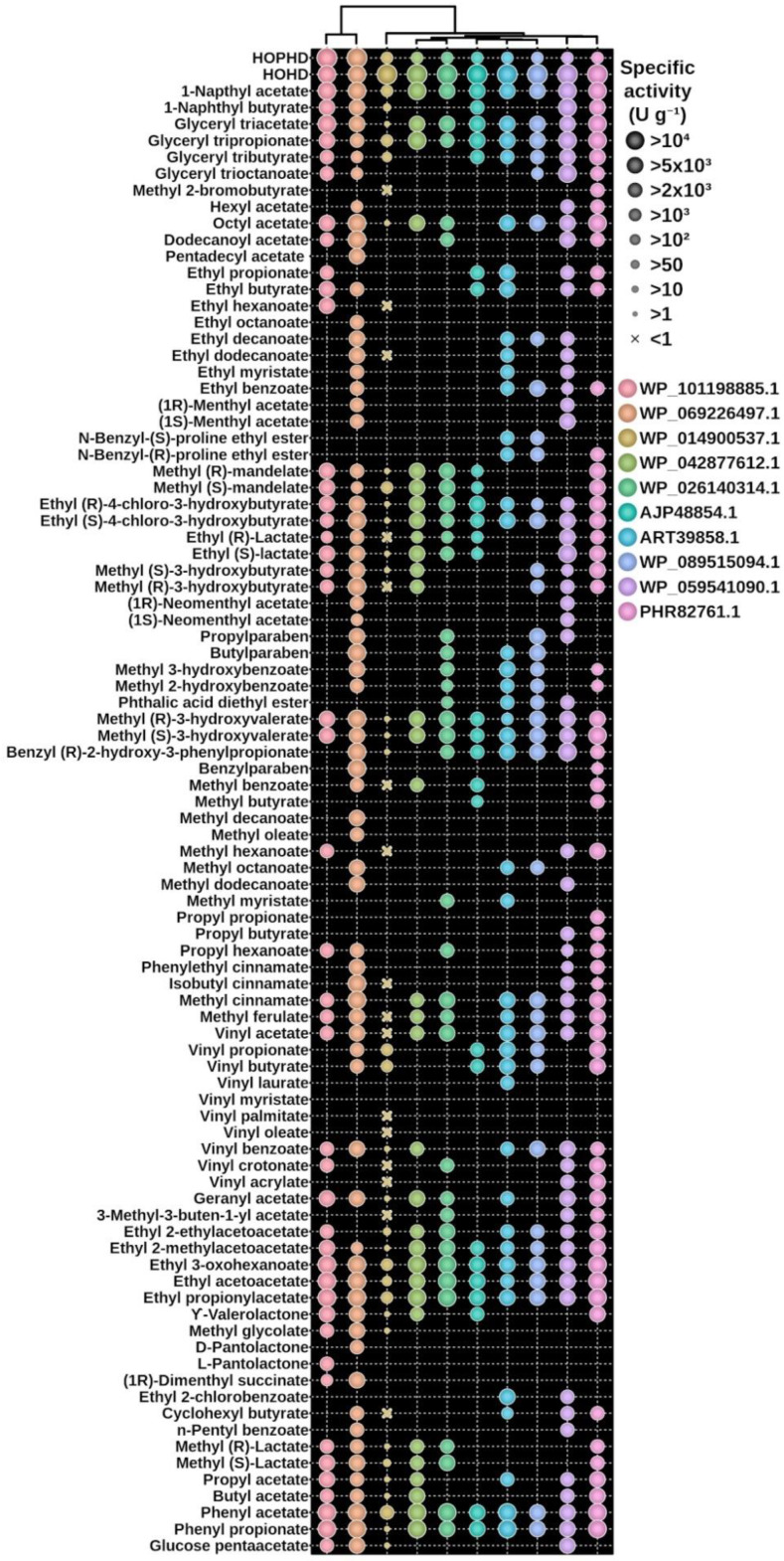
Substrate specificity of all ten hydrolases characterized in this study. The specific activity was measured for 2 meta-cleavage compounds (HOHD and HOPHD) and a set of 96 carboxylic esters (only those 89 found to be converted are shown). The proteins are organized based on their sequence similarity.

**Figure 5:**
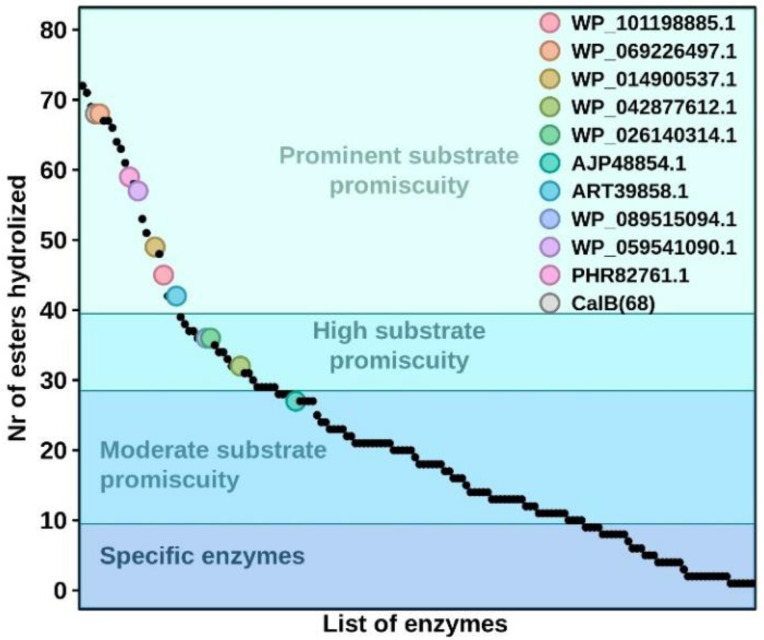
The ranking of all ten characterized hydrolases in terms of promiscuity. The number of substrates converted by each of the ten hydrolases herein reported compared to that of other 145 ester hydrolases previously characterized, using the same set of esters herein tested. This figure is created from data previously reported [4] and data herein reported for the ten hydrolases.

Once the esterase activity was confirmed, we further tested the hydrolytic activity towards a set of 96 structurally different esters based on Tanimoto-Combo similarity [4,39]. As shown in Figure 4, all enzymes were able to hydrolyze an ample set of esters, ranging from 27 (for AJP48854.1) to 68 (for WP_069226497.1). The specific activity ranges from 6. 50 (WP_069226497.1, being the most active) to 0.01 U mg^−1^ (WP_014900537.1, being the least active), depending on the substrate (Table S4). According to the criteria previously established [4], nine of the enzymes could be considered as having high-to-prominent substrate promiscuity as they hydrolyze 30 or more esters, whereas one (AJP48854.1) could be considered as moderately substrate promiscuous as it used less than 30 esters but more than 10 (a number below which an esterase could be considered substrate specific). Based only on the number of esters converted (Figure 5), these enzymes could be ranked among the hydrolases with the highest substrate promiscuity within a total of 145 esterases previously tested with a similar set of esters. Kinetic characterization using the model ester substrate *p*-NP propionate confirmed the high affinity of nine out the ten tested hydrolases for this substrate (*K*_m_ from14.5 to 48.7 µM) and the high conversion rates (*k*_cat_ from 2060 to 5043 min^−1^) of six of them (Table 2; Figure S1).

A database search indicated that all ten hydrolases showed from 60 to 82.9% identity with the meta-cleavage product hydrolase (MCP hydrolase) from *Pseudomonas fluorescens* IP01 (CumD) [40]. These hydrolases participate in the aerobic pathways for the bacterial degradation of aromatic carbons, in which aromatic compounds are cleaved into meta-ring fission compounds [35,38].

To check whether the ten proteins herein retrieved do show such activity, we tested two common meta-cleavage compounds 2-hydroxy-6-oxo-6-phenylhexa-2,4-dienoate (HOPHD) and 2-hydroxy-6-oxohepta-2,4-dienoate (HOHD) [38,40]. Using the standard assay conditions described in Section 2.11, we found all ten hydrolases converted HOPHD and HOHD, with HOHD being the preferred (from 132- to 273-fold) substrate for eight of the hydrolases (AJP48854.1, WP_042877612.1, WP_059541090.1, ART39858.1, WP_089515094.1, WP_026140314.1, PHR82761.1 and WP_014900537.1), and HOPHD the preferred (~2.5-fold) for two of them (WP_101198885.1 and WP_069226497.1) (Figure 4). The specific activity ranged from 23.2 to 0.4 U mg^−1^ for HOHD and from 89.04 to 0.8.6 U mg^−1^ for HOPHD, which indicates that both were the preferred substrates among all substrates tested (including non-activated and synthetic, activated *p*-NP esters).

When we compared the ten sequences to the training set, the average sequence identity dropped to 17%, with the maximum sequence identity being 67% and the minimum being 2% (Table S5). Additionally, if we compared the predicted sequences to the top 15 most promiscuous enzymes in the training set, which includes CalB, the average sequence identity dropped even further towards 15% with the maximum identity being 19% and the minimum identity being around 7%. So even if they have matching promiscuity their sequences are very different. In fact, sequence identity between those top 15 esterases is relatively low, with an average identity of 29%, a maximum identity of 51% and a minimum identity of 9%. Thus, as described before [4], sequence identity cannot be considered a predictor of promiscuity.

At the structural level, we did not find major differences when comparing the ten predicted sequences against the promiscuous or non-promiscuous training set, with a mean RMSD of 3.11 Å with the top 15 promiscuous esterases and 3.08 Å for the remaining of the training set (see, Table S7). Still, all ten selected enzymes share common active site physicochemical properties with the most promiscuous enzymes, derived from our filtering of the properties performed with SiteMap.

## 4. Conclusions

An essential aspect of implementing enzymes into industrial processes is the ability to perform fast and accurate bioprospecting. Modern genomic techniques are providing us with millions of new un-annotated sequences that have the potential of becoming new biocatalysts. However, techniques capable of working at a sequence level are necessary to cherry-pick those enzymes with favorable industrial qualities (or particular uses) in a feasible time. Here, ML methods seem an appropriate choice. As in any ML application, one of the main prerequisites is having enough (and diverse) data to train the model. We show in this study that if enough data is available, in our case a cross activity map between 145 enzymes and 96 substrates [4], a ML model can learn to distinguish those sequences encoding substrate promiscuity in ester hydrolases.

The experimental results herein provided, clearly demonstrate the validity of our ensemble classifier EP-pred in the prediction of substrate promiscuity of ester hydrolases. More importantly, the hydrolases herein retrieved were capable of hydrolyzing C-O bonds in an ample set of esters, but also C-C bonds in meta-cleavage products of catechol and biphenyl derivatives. Based on the sequence similarity and the substrate specificity and preferences, the enzymes herein retrieved could be classified as promiscuous MCP hydrolases with the ability to also convert a broad range of esters. This demonstrates the ability of the EP-pred system to identify hydrolases with the ability to catalyze ester hydrolysis for a broad range of different substrates even if it might not be the main reaction catalyzed by the enzymes.

The overall workflow applied in this project is not complex: (I) Filtering of sequence databases, such as a metagenomic database using HMM profiles to isolate ester hydrolases, (II) Prediction of substrate promiscuous ester hydrolases with EP-pred and (III) Homology modeling and structural analysis of the binding site cavity with SiteMap scores. Notice, that the workflow still uses molecular predictors to rank the final list of 100 sequences provided by the ML model; the sequence predictor is used as a means for selecting a short list of candidates, for which the structure generation and molecular descriptors extraction is an easy task. This was forced mainly by the academic (low budget) nature of our research. In a realistic industrial setting, hundreds of enzymes could be expressed and tested in vitro, thus possibly bypassing the last structural characterization step. Such an ML-only procedure would also facilitate the implementation of a similar approach for other enzymatic properties, as far as a comprehensive data training set is available; future studies will determine the wide applicability of this approach.

## Figures and Tables

**Table 1:** Specific activity against *p*-NP esters. The results are the mean ± SD of triplicates.

	Specific Activity (Units mg^−1^)		
Enzyme	*p*-NP Acetate	*p*-NP Propionate	*p*-NP Butyrate
AJP48854.1	2.96 ± 0.36	3.99 ± 0.25	1.68 ± 0.10
ART39858.1	2.53 ± 0.39	3.63 ± 0.26	1.48 ± 0.13
PHR82761.1	4.39 ± 0.44	2.75 ± 0.19	2.41 ± 0.25
WP_014900537.1	0.64 ± 0.02	2.19 ± 0.17	1.31 ± 0.18
WP_026140314.1	1.47 ± 0.05	3.33 ± 0.14	1.22 ± 0.09
WP_042877612.1	0.51 ± 0.01	2.57 ± 0.24	0.99 ± 0.06
WP_059541090.1	0.97 ± 0.02	2.96 ± 0.23	1.06 ± 0.08
WP_069226497.1	0.56 ± 0.01	2.26 ± 0.13	1.01 ± 0.04
WP_089515094.1	3.75 ± 0.17	4.46 ± 0.19	2.20 ± 0.15
WP_101198885.1	4.32 ± 0.14	5.85 ± 0.08	3.15 ± 0.25

**Table 2:** Kinetic parameters of selected hydrolases for *p*-NP propionate. The results are the mean ± SD of triplicates. Note: Kinetic parameters could not be determined (n.d.) because no reliable *K*_m_ could be determined (low affinity for the substrate under our experimental conditions).

Enzyme	*k*_cat_ (min^−1^)	*K*_m_ (µM)
AJP48854.1	n.d.	n.d.
ART39858.1	33.1 ± 0.1	33.3 ± 3.1
PHR82761.1	2569.3 ± 6.3	33.7 ± 3.6
WP_014900537.1	46.8 ± 0.0	16.9 ± 1.6
WP_026140314.1	5.9 ± 0.0	14.5 ± 1.1
WP_042877612.1	5043.0 ± 1268	65.8 ± 26.1
WP_059541090.1	3246.8 ± 10.6	31 ± 3.3
WP_069226497.1	3775.1 ± 37.2	46.5 ± 11.8
WP_089515094.1	2060.8 ± 5.7	23.5 ± 3.5
WP_101198885.1	3452 ± 15.9	48.7 ± 7.5

## Data Availability

The code, features and the results of the training can be downloaded from https://github.com/etiur/EP-pred.

## Supplementary Materials

The following supporting information can be downloaded at: https://www.mdpi.com/article/10.3390/biom12101529/s1, Figure S1: Calculation of kinetic parameters for *p*-NP propionate; Table S1: The features extracted from iFeature; Table S2: The features extracted from possum; Table S3: The sitemap scores of the 73 sequences that passed the first filter; Table S4: The activity assay of the ten enzymes versus the 96 ester substrates; Table S5: Pairwise sequence identity between the training set and the predicted sequences; Table S6: Quality of the models from ProsA-Web; Table S7: The RMSD of the ten sequences against the training set.
