# Supplementary material for: EP-Pred: A Machine Learning Tool for Bioprospecting Promiscuous Ester Hydrolases
Source: Biomolecules. 2022 Oct 21;12(10):1529. doi: 10.3390/biom12101529 (PMC9599548; doi:10.3390/biom12101529)
Supplement: Supplementary file 1 [file biomolecules-12-01529-s001.zip › biomolecules-1923580-supplementary_S1_S2_FS1.pdf]

# Supplementary information

Let’s define some abbreviations:

- aa: aminoacids
- L: Length of the sequences
- i: The ith residue of the sequence
- j: The column of a PSSM, which represent one of the 20 aa
- Pij: The PSSM score of amino acid j occurring at the position i of the sequence
- $\bar{P}$j: The mean of the scores over a column in the PSSM matrix.

## Ifeature features

**Table S1** | The features extracted from iFeature [19] used in this project

| iFeaure descriptors | Definition |
|---|---|
| Grouped amminoacid composition | aa composition and its variants count the % of each aa, dipeptide or tripeptide in the sequence. |
| Grouped dipeptide, tripeptide composition | |
| K-spaced aa group pairs composition | |
| Moran Autocorrelation | Autocorrelation is defined as the degree to which the observed value of a property at one region depends on values of the same property at neighboring regions separated by a predefined distance K. 8 aa properties are considered such as hydrophobicity scale, average flexibility index, polarizability parameter, etc. |
| Geary Autocorrelation | |
| Normalized Moreau-Broto Autocorrelation | |
| Composition | The 20 aa are divided into 3 groups for each of the 7 properties considered. For instance, residues can be divided into hydrophobic (CVLIMFW), neutral (GASTPHY), and polar (RKEDQN) groups and then it looks at the frequency of each group, how frequent are the transitions to other groups and the overall distribution along the sequences. |
| Transition | |
| Distribution | |
| Conjoint triad | It clusters the 20 aa into 7 classes and treats each tripeptide as a unit which results in a 3D matrix of 7X7X7 that counts the frequency of the possible combinations of classes in a unit. It counts the aa composition in a way that incorporates neighboring aa as well |
| K-spaced conjoint triad | |
| Sequence-order coupling number | These features try to incorporate sequence order information into the aa composition, we start with 20 components which are just the normal AAC and the rest of the entries in the vector are the different ranks. The ranks or sequence-coupling numbers, for instance, the first rank (let’s call it τ1) reflects the coupling effect between all the most contiguous residues, the second rank reflects the coupling effect of all residues separated by one residue and so on. The coupling effect for a predefined rank is determined as the normalized sum of the physicochemical distance between 2 residues separated by τ that was derived from the residue properties of hydrophobicity, hydrophilicity, polarity, and side-chain volume. There is a total of 400 such distance values. |
| Quasi sequence order | |
| Pseudo-aa-composition | Pseudo aa composition also incorporates sequence order information into the aa composition. It considers different tiers (let’s call it λ). For instance, the first tier reflects the sequence order information of all the most contiguous residues and the second tier represents the sequence order information between all residues separated by 1 residue and so on. This sequence order information is given by the normalized sum of a function that considers the relation between hydrophobicity, hydrophilicity and the side chain mass of residues separated by λ. |
| Amphiphilic Pseudo aa composition | |
| Pseudo K-tuple reduced aa composition type1 to 16 | |

## Possum Features

**Table S2** | The features extracted from possum [18]

<table>
<tr><th>Possum descriptors</th><th>Definition</th></tr>
<tr><td>AAC-PSSM</td><td>It scales the scores from the PSSM using a sigmoid function to the range from 0 to 1 and applies the logic of amino acid composition or AAC to the matrix. AAC counts the frequency of each of the 20 aa in the sequence. AAC-PSSM as such transforms the PSSM into a 20-dimensional vector where each component is average over the columns in PSSM, $\bar{P}$j.</td></tr>
<tr><td>D-FPSSM</td><td rowspan="2">It generates first a filtered PSSM by eliminating negative values and positive values that are greater than expected by random chance. It then sums all the probabilities in the same column of the new PSSM resulting in a vector of 20 dimensions. Each of the values in the vector is normalized using the min-max values of the entire dataset and the L of the sequence.</td></tr>
<tr><td>S-FPSSM</td></tr>
<tr><td>AB-PSSM</td><td rowspan="2">The protein sequence is divided into 20 equal blocks. Later, for each block, the row vectors of the PSSM matrix are added together and the final vector is divided by 5. Finally, the 20 vectors are placed side by side resulting in a feature vector of length 400.</td></tr>
<tr><td>RPM-PSSM</td></tr>
<tr><td>smoothed-PSSM window size (5)</td><td rowspan="3">Each row in the PSSM is represented by a vector of 20 dimensions containing the mutation scores. To consider the surrounding aa (upstream and downstream) they applied a sliding window of size w. The vectors of each row in the window are the sum of the vectors upstream and downstream, if w is 5 then it sums 2 vectors from upstream and 2 from downstream (The terminals are summed with vectors of zeros). Moreover, each row is represented as the concatenation of the upstream vectors and downstream vectors according to the window size.</td></tr>
<tr><td>smoothed-PSSM window size (7)</td></tr>
<tr><td>smoothed-PSSM window size (9)</td></tr>
<tr><td>PSSM-composition</td><td>For a PSSM of dimensions L X 20, it sums the scores that come from the same residues in L resulting in a 20 X 20 = 400-dimensional vector. For instance, the 20 scores of all the I (isoleucine) in the sequence, then this 20-dimensional vector is divided by the length of the sequence and scaled between [-1, 1]</td></tr>
<tr><td>DPC-PSSM</td><td>DPC-PSSM or dipeptide composition, similar to AAC-PSSM, applies the DPC logic to PSSMs after the scaling. There are 400 possible combinations of dipeptides which gives place to a 400-dimensional vector after the transformation. Each entry of the vector represents the average of the multiplication of probabilities or scores of each of every 2 contiguous residues in the sequence to mutate to each of the 400 dipeptide combinations.</td></tr>
<tr><td>k-separated-bigrams-PSSM</td><td>A bigram is a dipeptide, so like DPC-PSSM we have a 400-dimensional vector. K represents the distance between each bigram. However, each entry is only the sum of the multiplication of the probabilities of each bigram to mutate to each of the 400 possible dipeptide combinations. Moreover, the scores of PSSM are not scaled.</td></tr>
</table>

| | |
|---|---|
| tri-gram-PSSM | PSSM is not scaled. It applies the tripeptide composition logic to PSSM which results in a 20 x20 x 20 = 8000-dimensional vector. Each entry of the vector represents the sum of the multiplication of each of the residues in the tripeptide, for all tripeptides in the sequence, to each of the 8000 possible tripeptide combinations. |
| EEDP | It introduces the concept of the evolutionary difference formula (EDF). It calculates first the average mutation scores of 2 sets of contiguous residues being mutated to any of the 20 aa: [i-1, i], and [i, i+1]. Then it calculates the squared of their difference. The PSSM is transformed as a vector of 400 dimensions by applying the EDF which corresponds to all the possible dipeptides. For each dipeptide, it calculates the average of EDF of the contiguous residues to mutate to that dipeptide over the entire sequence. |
| TPC | The PSSM scores are scaled. It applies the transition probability matrix logic to the PSSM. Again, it is a 20 X 20 = 400-dimensional vector accounting for all the possible transitions. Each entry is a result of a division. For instance, for the dipeptide AA, the dividend is the sum of the multiplication of probabilities of each residue in a dipeptide in the sequence being mutated to A. The divisor is a sum of sums. We keep the first A invariant and change the second aa to all the other 19 aa (we have already calculated the AA transition) and for each of these dipeptides calculate the previous sum of probabilities but this time to the other transitions and sum all these 20 probabilities. |
| EDP | From the 400-dimensional matrix from EEDP, it derives a vector of 20 dimensions by averaging over the columns. |
| Pse-PSSM (ξ = 1) | It transforms the PSSM to a 20-dimensional vector, each entry being the average sum of all probabilities of a residue from the sequence to mutate to one of the 20 aa (average sum of the columns in a PSSM). Then, to incorporate sequence order information, they introduce ξ as the distance that separates 2 contiguous aa. For each ξ, it calculates 20 new values where each value is the average of all the squared differences between the probability of 2 contiguous residues being mutated to one of the 20 aa. |
| Pse-PSSM (ξ = 2) | |
| Pse-PSSM (ξ = 3) | |
| DP-PSSM | This feature connects two different vectors. The first one calculates and joints the average positive and negative values for each column resulting in a vector with a length of 40. The second contains the difference between the rows with a distance of k (k can have a value between 1 and 2). Then, it calculates the average square for the positive and negative differences. The length of the second feature vector will be 80; by merging with the first feature vector, the total feature vector length of 120 will be obtained. |
| PSSM-AC | They apply the autocross-covariance (ACC) transformation to PSSM, which considers the correlation between pairs of aa separated by a distance lg. Therefore, we would have 20 dipeptides with the same aa (AC) and 380 with |
| PSSM-CC | |

| | |
|---|---|
| | different aa (CC). The correlation is calculated as the average of (Pi,j - $\bar{P}$j) · (Pi+lg,j - $\bar{P}$j) along the sequence. |
| RPSSM | It reduces the columns of the PSSM from 20 to 10 aa according to their properties. The new columns are just the averages of the scores from the same group. Then it transforms the PSSM into a vector of 10 dimensions where each dimension is the variance of the probabilities. First, the mean of probabilities for each column in PSSM are calculated, then the squared difference between the individual values in the column and the mean for that column is computed and summed. |

ART39858.1

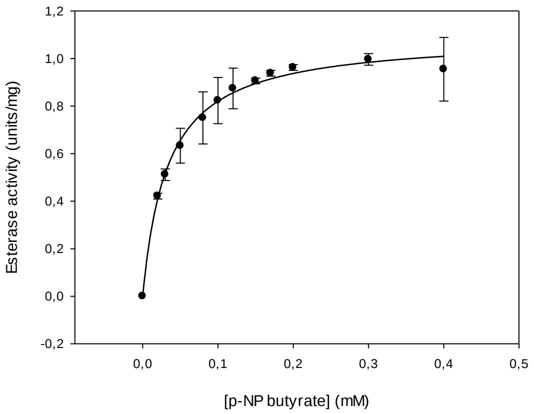


PHR82761.1

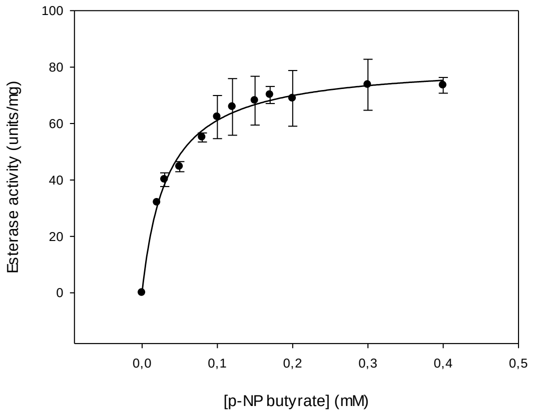


WP_014900537.1

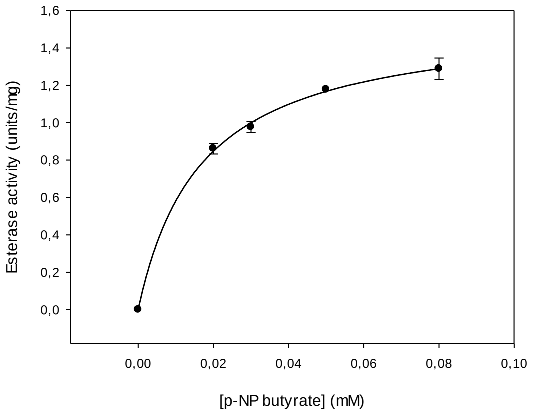


WP_026140314.1

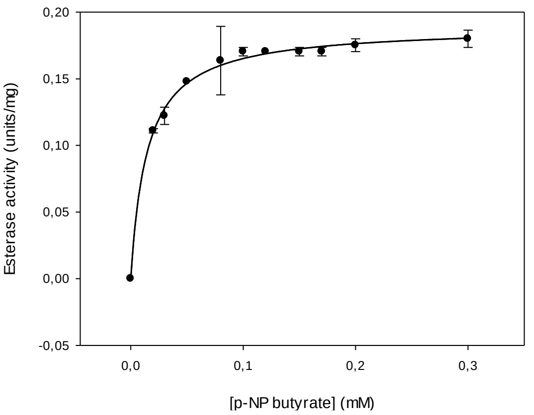

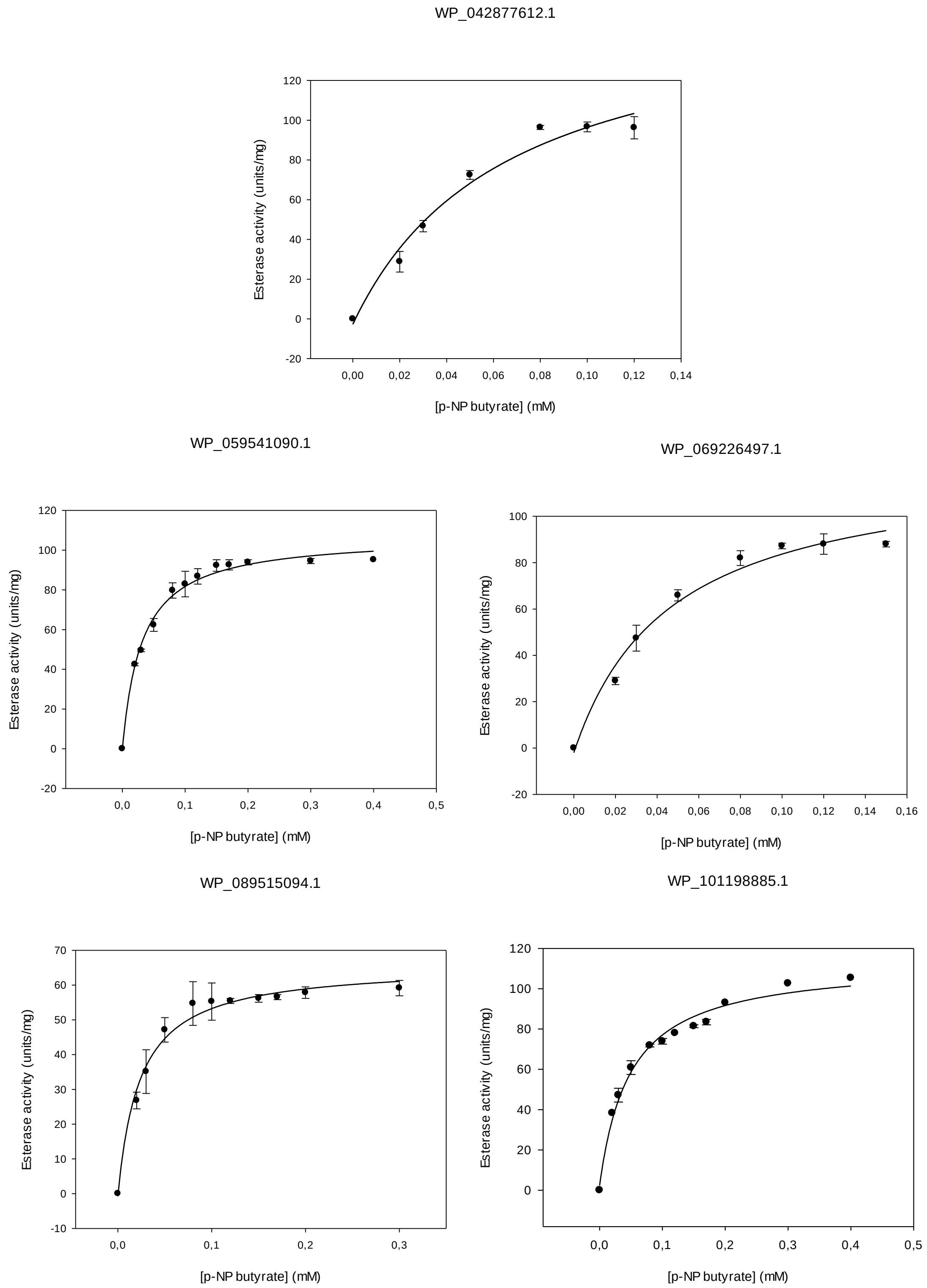


**Figure S1**| Calculation of kinetic parameters. Data obtained based on the methods detailed in the Materials and Methods section. Regression performed using SigmaPlot 14.0 software.

Raw data on the kinetics of the enzymes which gave place to the plots

| **ART39858.1** | | |
|---|---|---|
| [p-NP propionate] (mM) | SPEC. ACT. (U/mg) | STAND. DEV. |
| 0 | 0 | 0 |
| 0,02 | 0,421 | 0,012 |
| 0,03 | 0,512 | 0,024 |
| 0,05 | 0,633 | 0,073 |
| 0,08 | 0,750 | 0,110 |
| 0,1 | 0,823 | 0,097 |
| 0,12 | 0,874 | 0,085 |
| 0,15 | 0,906 | 0,012 |
| 0,17 | 0,938 | 0,012 |
| 0,2 | 0,962 | 0,012 |
| 0,3 | 0,996 | 0,024 |
| 0,4 | 0,955 | 0,134 |

| **PHR82761.1** | | |
|---|---|---|
| [p-NP propionate] (mM) | SPEC. ACT. (U/mg) | STAND. DEV. |
| 0 | 0,000 | 0,000 |
| 0,02 | 31,991 | 0,000 |
| 0,03 | 40,109 | 2,411 |
| 0,05 | 44,731 | 1,809 |
| 0,08 | 55,060 | 1,608 |
| 0,1 | 62,294 | 7,636 |
| 0,12 | 65,911 | 10,047 |
| 0,15 | 68,122 | 8,641 |
| 0,17 | 70,131 | 3,014 |
| 0,2 | 68,925 | 9,846 |
| 0,3 | 73,748 | 9,043 |
| 0,4 | 0,000 | 0,000 |

| **WP_014900537.1** | | |
|---|---|---|
| [p-NP propionate] (mM) | SPEC. ACT. (U/mg) | STAND. DEV. |
| 0 | 0,000 | 0,000 |
| 0,02 | 0,861 | 0,029 |
| 0,03 | 0,976 | 0,029 |
| 0,05 | 1,177 | 0,000 |
| 0,08 | 1,289 | 0,057 |

| **WP_026140314.1** | | |
|---|---|---|
| [p-NP propionate] (mM) | SPEC. ACT. (U/mg) | STAND. DEV. |
| 0 | 0,000 | 0,000 |
| 0,02 | 0,111 | 0,002 |
| 0,03 | 0,122 | 0,006 |
| 0,05 | 0,148 | 0,000 |
| 0,08 | 0,164 | 0,026 |

| | | |
|---|---|---|
| 0,1 | 0,170 | 0,003 |
| 0,12 | 0,170 | 0,000 |
| 0,15 | 0,170 | 0,003 |
| 0,17 | 0,170 | 0,003 |
| 0,2 | 0,175 | 0,005 |
| 0,3 | 0,180 | 0,006 |

| **WP_042877612.1** | | |
|---|---|---|
| [p-NP propionate] (mM) | SPEC. ACT. (U/mg) | STAND. DEV. |
| 0 | 0,000 | 0,000 |
| 0,02 | 28,792 | 5,205 |
| 0,03 | 46,712 | 2,833 |
| 0,05 | 72,473 | 2,240 |
| 0,08 | 96,389 | 0,988 |
| 0,1 | 96,653 | 2,438 |
| 0,12 | 96,258 | 5,600 |

| **WP_059541090.1** | | |
|---|---|---|
| [p-NP propionate] (mM) | SPEC. ACT. (U/mg) | STAND. DEV. |
| 0 | 0,000 | 0,000 |
| 0,02 | 42,440 | 0,643 |
| 0,03 | 49,514 | 0,643 |
| 0,05 | 62,374 | 3,215 |
| 0,08 | 79,736 | 3,858 |
| 0,1 | 82,952 | 6,430 |
| 0,12 | 86,810 | 3,858 |
| 0,15 | 92,276 | 2,894 |
| 0,17 | 92,597 | 2,572 |
| 0,2 | 93,883 | 1,286 |
| 0,3 | 94,526 | 1,286 |
| 0,4 | 95,169 | 0,000 |

| **WP_069226497.1** | | |
|---|---|---|
| [p-NP propionate] (mM) | SPEC. ACT. (U/mg) | STAND. DEV. |
| 0 | 0,000 | 0,000 |
| 0,02 | 28,937 | 1,608 |
| 0,03 | 47,424 | 5,627 |
| 0,05 | 65,911 | 2,411 |
| 0,08 | 81,987 | 3,215 |
| 0,1 | 87,212 | 1,206 |
| 0,12 | 88,015 | 4,421 |
| 0,15 | 88,015 | 1,206 |

| **WP_089515094.1** | | |
|---|---|---|
| [p-NP propionate] (mM) | SPEC. ACT. (U/mg) | STAND. DEV. |
| 0 | 0,000 | 0,000 |
| 0,02 | 26,793 | 2,402 |
| 0,03 | 35,108 | 6,283 |

| | | |
|---|---|---|
| 0,05 | 47,119 | 3,511 |
| 0,08 | 54,695 | 6,283 |
| 0,1 | 55,249 | 5,359 |
| 0,12 | 55,434 | 0,739 |
| 0,15 | 56,173 | 1,109 |
| 0,17 | 56,543 | 0,739 |
| 0,2 | 57,836 | 1,663 |
| 0,3 | 59,130 | 2,217 |

| **ART39858.1** | | |
|---|---|---|
| [p-NP propionate] (mM) | SPEC. ACT. (U/mg) | STAND. DEV. |
| 0 | 0,000 | 0,000 |
| 0,02 | 38,308 | 0,000 |
| 0,03 | 47,202 | 3,420 |
| 0,05 | 60,883 | 3,420 |
| 0,08 | 71,828 | 0,684 |
| 0,1 | 73,881 | 1,368 |
| 0,12 | 77,985 | 0,000 |
| 0,15 | 81,406 | 0,684 |
| 0,17 | 83,458 | 1,368 |
| 0,2 | 93,035 | 0,000 |
| 0,3 | 102,612 | 0,000 |
| 0,4 | 105,348 | 0,000 |
